# Beyond the Green Light: Evaluating the Uncertainties of Resmetirom for Non‐Cirrhotic Nonalcoholic Steatohepatitis Treatment

**DOI:** 10.1002/hsr2.71613

**Published:** 2025-12-30

**Authors:** Hafiz Muhammad Moaaz Sajid, Fakiha Rahman, Abubakr Mahmoud

**Affiliations:** ^1^ Punjab Medical College Faisalabad Medical University Faisalabad Pakistan; ^2^ King Edward Medical University Lahore Pakistan; ^3^ University of Khartoum Khartoum Sudan

Dear Editor,

We read with great interest the recent perspective by Hasan et al. highlighting the US Food and Drug Administration (FDA) approval of resmetirom for non‐cirrhotic nonalcoholic steatohepatitis (NASH) with moderate to advanced fibrosis (F2–F3) [1]. This marks an important milestone in the pharmacological management of a condition for which lifestyle modification and bariatric surgery have historically been the primary options. We concur that the advent of resmetirom offers a long‐awaited alternative, especially for patients struggling with weight loss and adherence to lifestyle interventions.

The approval is underpinned by robust phase 3 trial data showing that resmetirom significantly increased rates of NASH resolution without fibrosis progression and improved fibrosis stage compared with placebo at 52 weeks [2]. Additionally, recent meta‐analyses confirm reductions in hepatic fat content and improvements in liver enzymes, with an overall favourable safety profile [3]. These findings are encouraging, and resmetirom's oral administration and once‐daily dosing may enhance long‐term adherence compared to intensive lifestyle programmes alone.

However, several concerns warrant consideration before declaring resmetirom a transformative solution. First, while histological endpoints in clinical trials are promising, the durability of these improvements beyond 1 year and their impact on clinically meaningful outcomes, such as cirrhosis, hepatocellular carcinoma, liver‐related mortality, and transplant rates, remain unknown. Long‐term extension studies and post‐marketing surveillance are essential to confirm sustained benefit and safety.

Second, pivotal trials excluded patients with cirrhosis, advanced comorbidities, or poorly controlled metabolic disease [2]. These exclusions limit the generalisability of the data to the real‐world NASH population, where late presentation and multimorbidity are common. There is a need for pragmatic trials and registry‐based studies evaluating resmetirom in these high‐risk cohorts.

Third, access and affordability pose significant barriers. NASH is prevalent worldwide, with increasing incidence in low‐ and middle‐income countries (LMICs) due to rising obesity and type 2 diabetes rates [4]. If resmetirom pricing follows patterns seen with other novel therapeutics, its uptake in resource‐limited settings may be minimal, exacerbating global health disparities. This underscores the need for transparent pricing strategies, inclusion in essential medicines lists, and public‐private partnerships to ensure equitable access.

Fourth, the safety profile, though largely favourable, includes gastrointestinal adverse events, most commonly nausea and diarrhoea, occurring more frequently than with placebo [2, 3]. While not typically severe, these can affect quality of life and adherence, especially in populations already burdened by polypharmacy. Careful monitoring and patient education are vital in mitigating these issues.

Finally, while resmetirom represents a significant advance, it should complement rather than replace foundational lifestyle interventions. Weight loss through dietary change and physical activity improves hepatic steatosis, inflammation, and fibrosis while also reducing cardiovascular risk [5]. Combining pharmacotherapy with structured behavioural programmes may yield synergistic benefits and address the metabolic drivers of NASH more comprehensively.

In conclusion, resmetirom's approval is a welcome and important development in NASH therapeutics. Yet its optimal integration into clinical practice requires addressing gaps in long‐term efficacy data, expanding evidence to real‐world populations, ensuring affordability and access, and reinforcing the central role of lifestyle modification. As the global burden of NASH continues to rise, a multifaceted approach, balancing pharmacological innovation with preventive strategies, will be essential to achieving sustainable reductions in disease progression and associated mortality.

## Author Contributions

**Hafiz Muhammad Moaaz Sajid:** conceptualization; writing – original draft; writing – review and editing; data curation; supervision; project administration. **Fakiha Rahman:** writing – original draft; methodology; investigation; validation. **Abubakr Mahmoud:** writing – original draft; writing – review and editing; methodology.

## Data Availability

Data sharing not applicable to this article as no datasets were generated or analysed during the current study.
